# Deep Learning Pipeline for Automated Assessment of Distances Between Tonsillar Tumors and the Internal Carotid Artery

**DOI:** 10.1002/hed.28200

**Published:** 2025-06-03

**Authors:** Aseem Jain, Ameen Amanian, Nimesh Nagururu, Francis X. Creighton, Eitan Prisman

**Affiliations:** ^1^ Department of Otolaryngology‐Head and Neck Surgery University of Iowa Iowa City Iowa USA; ^2^ Division of Otolaryngology‐Head and Neck Surgery University of British Columbia Vancouver British Columbia Canada; ^3^ Department of Otolaryngology‐Head and Neck Surgery Johns Hopkins Medical Institute Baltimore Maryland USA

**Keywords:** deep learning, internal carotid artery, medical imaging, tonsillar carcinoma, transoral robotic surgery

## Abstract

**Background:**

Evaluating the minimum distance (dTICA) between the internal carotid artery (ICA) and tonsillar tumors (TT) on imaging is essential for preoperative planning; we propose a tool to automatically extract dTICA.

**Methods:**

CT scans of 96 patients with TT were selected from the cancer imaging archive. nnU‐Net, a deep learning framework, was implemented to automatically segment both the TT and ICA from these scans. Dice similarity coefficient (DSC) and average hausdorff distance (AHD) were used to evaluate the performance of the nnU‐Net. Thereafter, an automated tool was built to calculate the magnitude of dTICA from these segmentations.

**Results:**

The average DSC and AHD were 0.67, 2.44 mm, and 0.83, 0.49 mm for the TT and ICA, respectively. The mean dTICA was 6.66 mm and statistically varied by tumor T stage (*p* = 0.00456).

**Conclusion:**

The proposed pipeline can accurately and automatically capture dTICA, potentially assisting clinicians in preoperative evaluation.

## 1 Introduction

Since described in 2006, transoral robotic surgery (TORS) has become a widely adopted technique for resection of early stage (T1 and T2) tonsillar carcinoma [1]. Compared to traditional, open techniques, minimally invasive methods including TORS offer distinct advantages including avoidance of an external incision and greater preservation of normal anatomy leading to better functional outcomes [2]. TORS, additionally, belays technical benefits with improved range of motion, tremor reduction, and view magnification [3]. Other groups have shown that these advantages translate into decreased rates of gastrostomy and tracheostomy tubes and overall shorter hospital stays [4].

Although rare, one of the feared complications of TORS is injury to critical vasculature in the parapharyngeal space (PPS). As described in the literature, the PPS is a triangular space that borders from the skull base to the greater cornu of the hyoid bone and includes the internal carotid artery (ICA) and branches of the external carotid artery (ECA) [5, 6]. Specifically, injury to the ICA could lead to life‐threatening surgical complications [6]. While the risk for severe injury is rare, most TORS procedures involve working within the PPS [5]. Additionally, while relatively uncommon (3%–10%), anatomic variants of the ICA such as a retropharyngeal ICA reduce the distance between the tumor border and the ICA, therefore making this variation a relative contraindication for TORS [7, 8]. Similarly, tumors that are adjacent to the carotid bulb or ICA increase the risk of carotid exposure during surgery and should not undergo this minimally invasive approach [9]. Given these risks and complexities, pre‐surgical imaging analysis is critical for both the identification of appropriate candidates for TORS and pre‐operative planning [8].

Evaluating the appropriateness of TORS pertinent to a patient's anatomy traditionally involves qualitative analysis of imaging without strict quantitative measurements. The lack of objective analysis is likely due to the technical complexity of obtaining such measurements. Manual two‐dimensional (2D) measurement of the minimum distance between the tumor and the ICA (dTICA) can be time‐consuming to capture from CT scans and can be inaccurate in the setting of deciphering from a three‐dimensional structure. Further, as with any manual annotation process, measuring distances can be subject to interrater variability, often requiring multiple raters to verify the accuracy [10]. On the other hand, 3D measurements, while more accurate, are difficult to acquire as they require time‐intensive manual segmentations of the 3D structures from CT scans. As others have noted, performing large‐scale segmentations manually is both impractical and, similar to manual measurements, subject to interrater variability [10, 11]. However, with the advent of deep learning (DL), researchers can automatically segment and subsequently produce 3D structures including the tumor and its margins, and the ICA from unstructured CT scans [12, 13]. This paper describes a deep learning pipeline able to (1) automatically segment and produce 3D models of tonsillar tumors and ICAs from CT scans, and (2) use these segmentations to automatically extract the minimum distance between the tumor and the ICA (dTICA). To the best of our knowledge, this pipeline represents a novel end‐to‐end application of deep learning integration for the assessment of tonsillar carcinomas.

## 2 Methods

### 2.1 Data

Non‐contrast CT scans were obtained from The Cancer Imaging Archive, *Radiomics outcomes prediction in Oropharyngeal cancer* dataset, which contains pre‐segmented tumor and nodal volume for oropharyngeal cancers [14]. These segmentations were performed by expert radiologists and were independently verified. The 96 scans of individuals with tonsillar cancer were extracted from the dataset for this investigation. As data from the Cancer Imaging Archive is in the public domain, no IRB approval was sought. CT scans used for this study had a resolution of 512×512 and comprised 1 mm slices.

While tonsillar tumor segmentations were provided with the dataset, no ICA segmentations were available. Accordingly, the ICA was manually segmented bilaterally from the carotid bifurcation to the mandibular condyle in a subset of 60 scans by AJ. This subset was created with an even distribution of tumor T stages from T1 to T4 (*n* = 15 per stage) to assess how dTICA changes across T stage. The ICA was not segmented past these landmarks as the closest point on the ICA to the tumor is usually found within these bounds. The accuracy of the ICA segmentations and associated 3D volumes was independently verified by two otolaryngologists (FC, EP).

### 2.2 Segmentation Network

**FIGURE 1:**
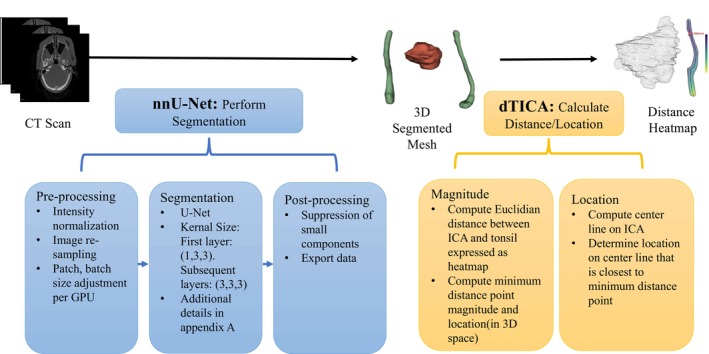
Overview of the method to (1) segment Tonsil Tumor (red) and internal carotid artery (green) and (2) compute the distance heatmap between them. Additional details of the segmentation network architecture provided in Supporting Information Appendix S1. [Color figure can be viewed at wileyonlinelibrary.com]

Figure 1 provides an overview of the proposed pipeline. Briefly, the pipeline first segments the CT scans to generate 3D models of the tumor and ICA. The pipeline then calculates the dTICA and its location at this point on the carotid artery. The DL network, nnU‐Net, a widely adopted deep learning semantic segmentation network in medical image analysis, was chosen to segment and generate 3D models of the tonsillar tumors and ICAs from CT scans [13, 15, 16, 17, 18]. More details about the usage of nnU‐Net are described in the discussion section.

The ICA and tonsillar tumor segmentations were trained via separate 3D nnU‐Nets so that the tonsillar tumor network could take advantage of the entire 96 scan dataset, while the ICA network employed the 60 scans with ICA segmentations generated in this investigation. The networks were trained and evaluated with standard fivefold cross validation for 100 epochs on an Nvidia Quadro RTX 8000 graphics processing unit. Given nnU‐Net's ability to automatically optimize hyperparameters, other deep learning variables were not adjusted by the authors. Other default parameters for 3D segmentation using nnU‐Net were: patch size [64, 160, 160], spacing [2, 0.69, 0.69], and batch size [2]. The framework involved (1) randomly splitting the data into training and validation splits (80% for training, 20% for validation), (2) using the training split to generate the DL model, and then (3) evaluating the validation split. This process was repeated for a total of five cycles.

To quantify the performance of the deep learning framework, we utilized multiple metrics to compare the DL predicted segmentation and the ground truth manual segmentation. We choose to use average hausdorff distance (AHD) and dice similarity coefficient (DSC) to estimate the surface and volume error respectively between the generated inferences and the ground truth. From the 3D segmentations generated by the nnU‐Net model, AHD was calculated as the average distance in millimeters (mm) between each surface point in the ground truth model and its closest point in the predicted model. The DSC was also calculated, which determines the volumetric overlap between two structures. A DSC score of 1 represents perfect overlap between the ground truth and the generated label [19]. All deep learning analysis was done in Python—version 3.11.3.

### 2.3 Distance From Tumor to ICA (dTICA) Measurement Tool

**FIGURE 2:**
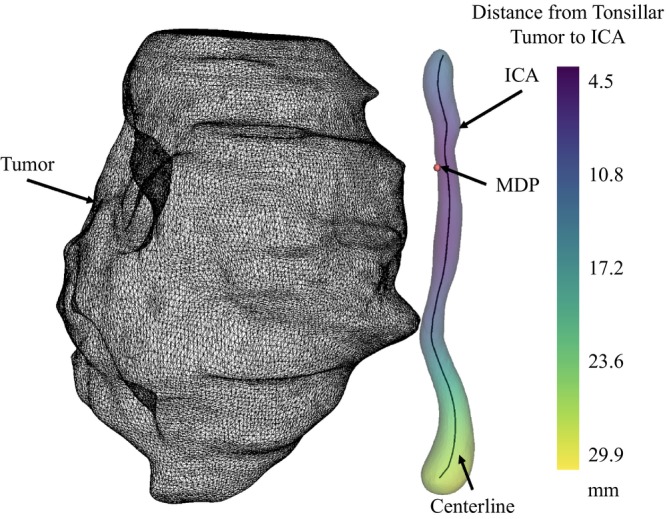
View of minimum distance heatmap between tumor (black) and internal carotid artery (ICA). Minimum distance point (MDP) and center line are labeled. [Color figure can be viewed at wileyonlinelibrary.com]

To extract dTICA from the segmentations generated by the DL framework, an automated tool was developed using a combination of Python and open‐source packages (model‐to‐model distance and vascular modeling tool kit) within 3D Slicer [20, 21, 22]. The segmented tumor and ICA were first converted from voxel‐based segmentations into mesh point cloud files. The model‐to‐model distance sub‐package, which calculates the Euclidean distance, *v*, was calculated between two mesh point clouds in 3D Slicer. The vector *v* is calculated by

$$v=\sqrt{\left({\left(m_{1x}-m_{2x}\right)}^{2}+{\left(m_{1y}-m_{2y}\right)}^{2}+{\left(m_{1z}-m_{2z}\right)}^{2}\right)}$$

where *m*_1_ and *m*_2_ correspond to the external surface points of the two mesh point clouds. The magnitude of vector v is used to generate a color map. In our case, the color of each point on the mesh represented the minimum distance between that ICA point and the tumor mesh [21]. A detailed visualization of this heatmap is seen in Figure 2; darker colors on the ICA correspond to decreased distance between the two structures. The minimum magnitude of heatmap values was automatically recorded as the dTICA. The dTICA was also manually measured from the 3D models using the annotation tool within 3D Slicer for further validation of the pipeline and assessing the performance of the automated tool.

Figure 2 also illustrates the minimum distance point (MDP)—the point on the ICA corresponding to the dTICA measurement. The location of the MDP was quantified as its distance to the carotid bifurcation along the path of the ICA centerline. The centerline (shown in Figure 2) through the ICA was computed using the vascular modeling tool kit (VMTK) sub‐package in 3D Slicer. This package automatically extracted the centerline by finding a point within a slice of a 3D model that was equally distant to the boundary then iterating through each slice of the model [22].

### 2.4 Tumor Measures

**FIGURE 3:**
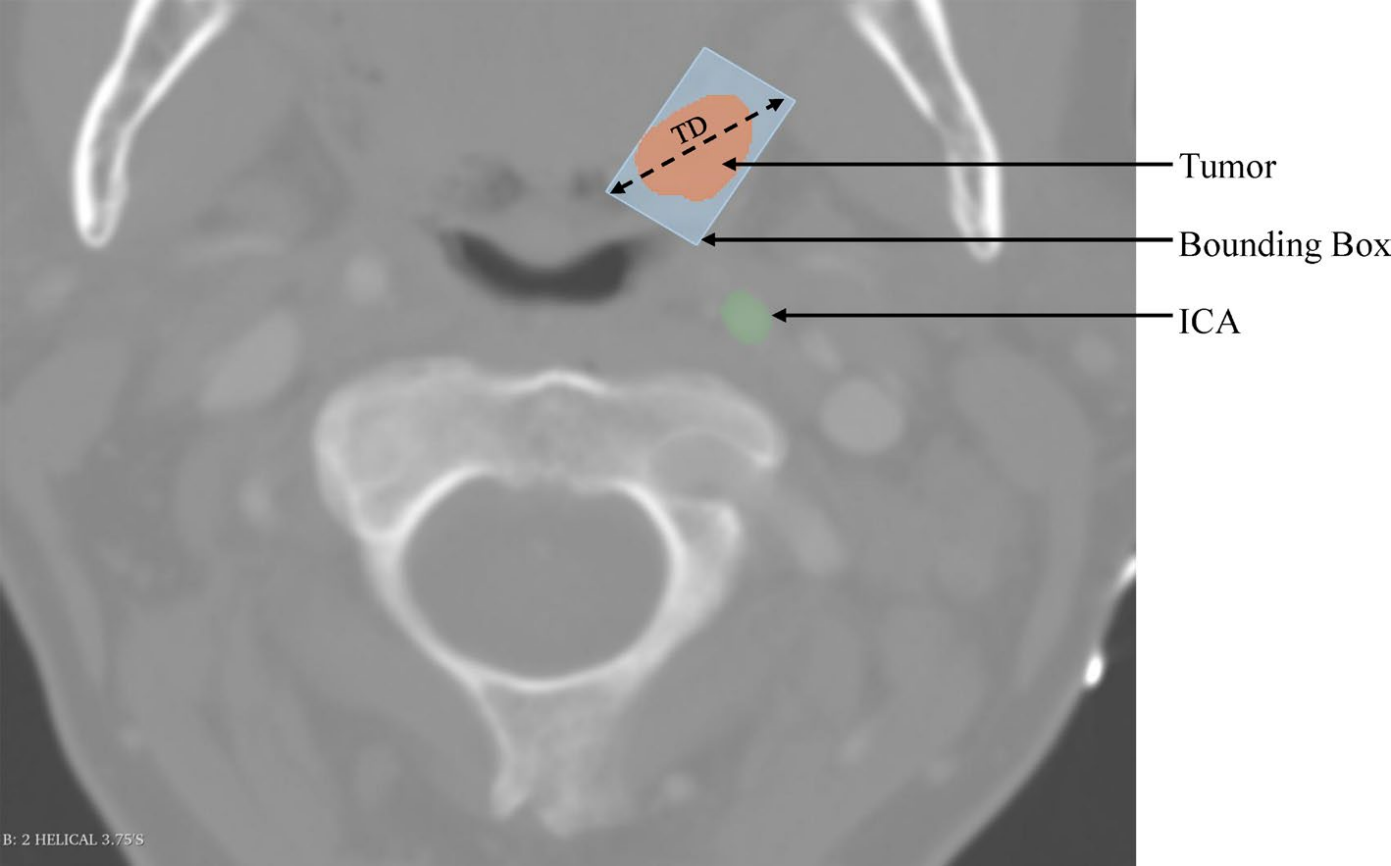
CT scan of tonsillar tumor (orange) and internal carotid artery (ICA, green). Bounding box (light blue) around tumor with transverse plane diameter (TD, dashed arrow) are depicted. [Color figure can be viewed at wileyonlinelibrary.com]

From the tumor meshes, tumor parameters such as volume, surface area (SA), and transverse‐plane diameter (TD) were also automatically extracted using the segment statistics tool within 3D slicer. As depicted in Figure 3, TD was computed by determining the smallest bounding box around the tumor and then calculating the hypotenuse along the transverse plane of this box. TD was captured to assess if the diameter measured in 3D could be useful in the clinical assessment of dTICA.

### 2.5 Statistical Analysis

For each tonsillar tumor in the subset of 60 scans with both tonsillar tumor and ICA segmentations, we computed volume, surface area, TD, magnitude of the dTICA, and the location of MDP along the carotid artery. Clinical data including stage, patient age, smoking year, etc. was also captured. Relationships between tumor stage, volume, surface area, and TD with dTICA and MDP location were evaluated with ANOVAs and pairwise *t*‐tests for categorical variables and Pearson's correlation coefficient for continuous variables. Tumor characteristics were compared among T stages with ANOVA. All statistical analyses were conducted using Matlab Version 2023A, with an alpha level of 0.05.

## 3 Results

Table 1 shows the distribution of patient characteristics used for the dataset of 60 CT scans that were used to compare how the tumor stage impacted dTICA. This dataset was comprised primarily of male patients (76.6%) diagnosed in their late 50s (mean age 57.2, standard deviation 8.4) with an average 22.3‐pack year smoking history. Most patients in this dataset were primarily treated with a combination of induction chemotherapy and concurrent chemoradiotherapy (24/60) [14].

**TABLE 1:** Demographic data of sample population.

Total	60
Gender	
Male	46
Female	14
Average age at diagnosis	57.2
Average smoking pack years	21.5
Treatment type	
Radiation alone	8
Induction chemotherapy + radiation alone	10
Induction chemotherapy + concurrent chemoradiotherapy	24
Concurrent chemoradiotherapy	18

**FIGURE 4:**
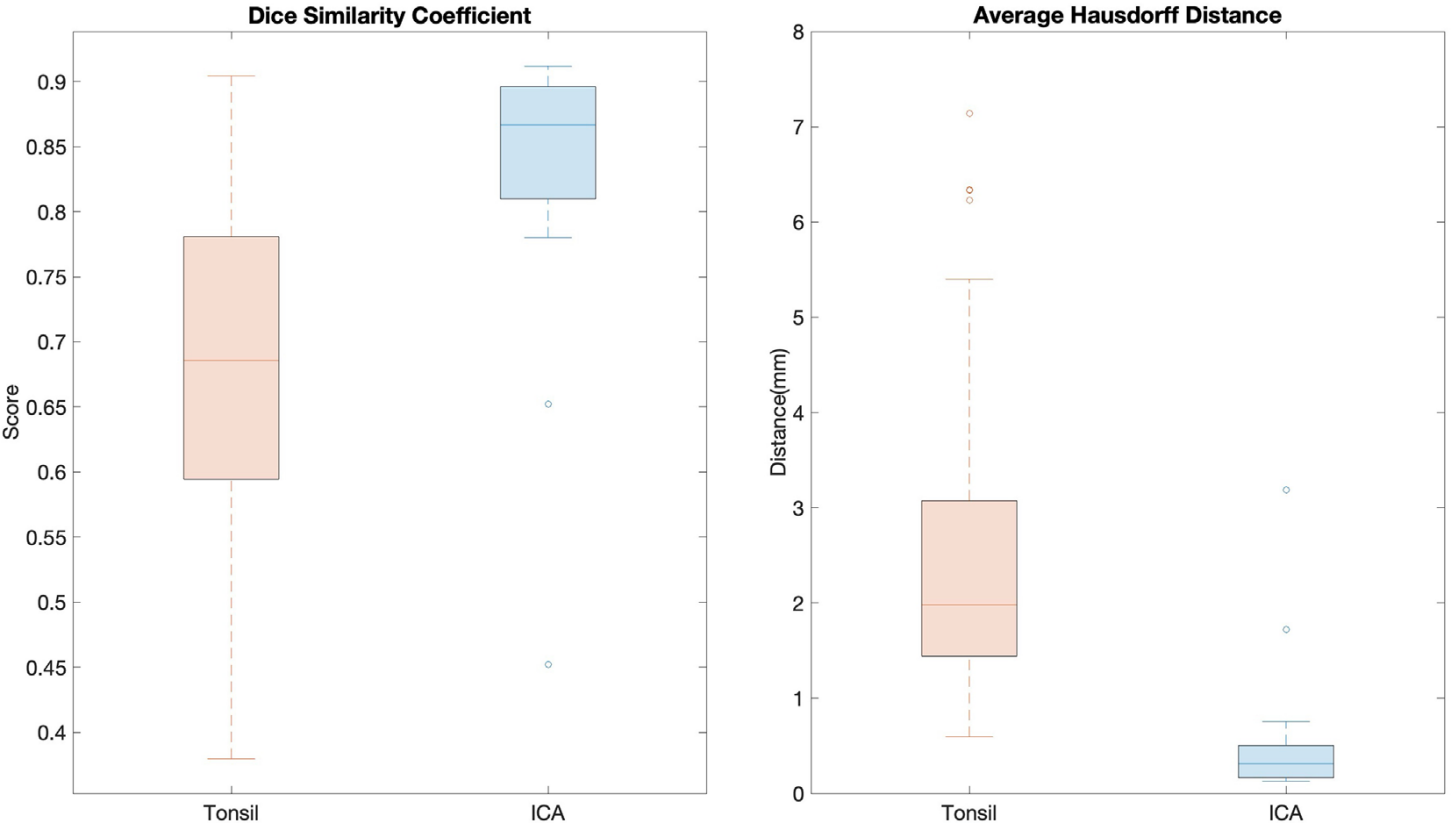
(Left) Average hausdorff distances between the “Ground Truth” segmentations and “Predicted” segmentations for tonsillar tumor and ICA. (Right) Dice similarity coefficients between the “Ground Truth” segmentations and “Predicted” segmentations for tonsillar tumor and ICA. [Color figure can be viewed at wileyonlinelibrary.com]

The performance metrics assessed via the DL framework can be visualized in Figure 4. AHD for tonsil tumor segmentation was 2.44 ± 1.42 mm and 0.49 ± 0.67 mm for ICA segmentation. Similarly, average DSC scores were 0.67 ± 0.14 and 0.83 ± 0.10 for the tonsillar tumor and ICA segmentations, respectively. The total training time using the fivefold cross‐validation described above for the nnU‐Net was around 450 min using a Quadro RTX 8000 GPU with 48GB of VRAM. The inference of the validation scans (i.e., automated segmentation) took on average 90 s per scan.

**FIGURE 5:**
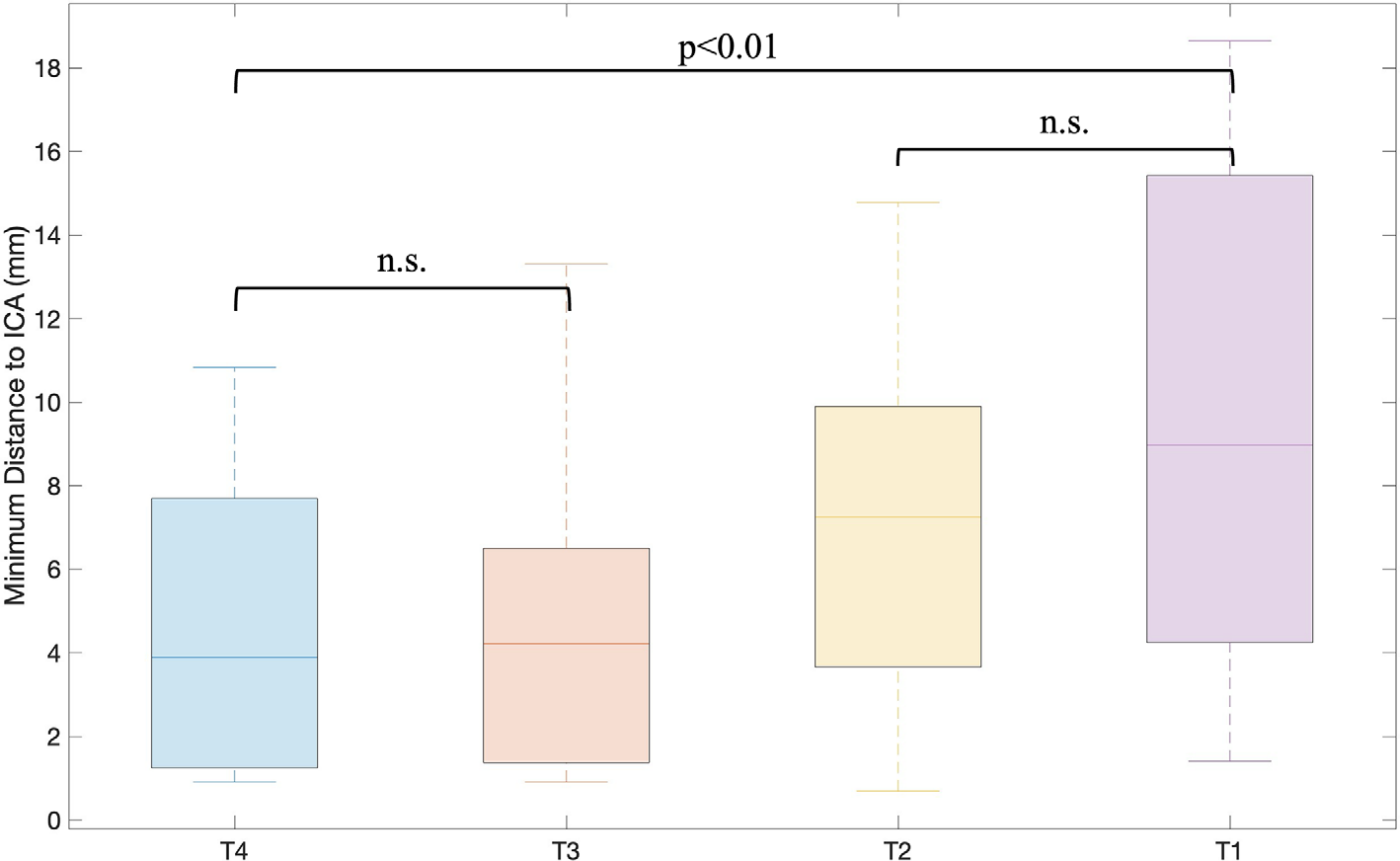
Minimum distance to ICA by tumor T stage. No statistical difference (n.s.) in dTICA was observed between stages T1 and T2; a similar trend was found between T3 and T4. A statistical difference was noted between stages T4/T3 and T2/T1. [Color figure can be viewed at wileyonlinelibrary.com]

**FIGURE 6:**
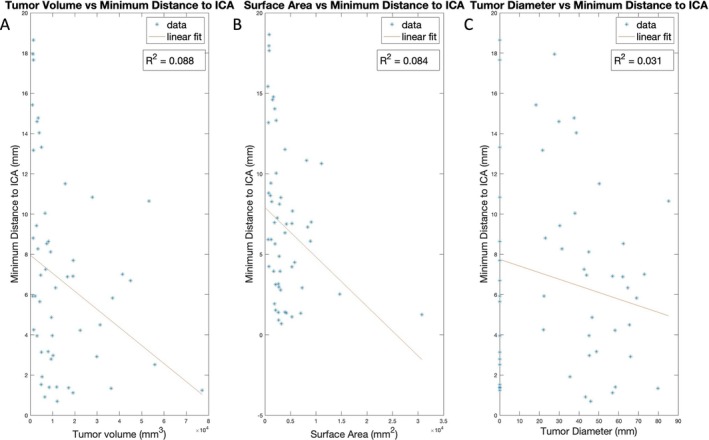
Minimum distance to ICA by (A) tumor volume, (B) surface area, and (C) tumor diameter. Tumor diameter corresponds to the diameter calculated from the bounding box in the transverse plane (TD). [Color figure can be viewed at wileyonlinelibrary.com]

As mentioned above, to extend this deep learning framework, a tool to extract the minimum distance between tumor and ICA (dTICA) was created and applied to the smaller subset of 60 scans. The average dTICA for this dataset was 6.65 mm (std 4.93 mm). Figure 5 demonstrates how dTICA differs across tumor T stage (Figure 5). The average dTICA was 4.80, 4.75, 7.49, and 9.76 mm for T4, T3, T2, and T1 stage cancer respectively. Comparing dTICA across all four T stages showed a statistical difference between all four groups (*p* = 0.01). The greatest difference was between tumors staged less than or equal to T2 and greater than T2 (*p* < 0.01). There was no statistical difference between T1 versus T2 or T3 versus T4 stages when comparing dTICA. The relationships between tumor volume, surface area, TD, and dTICA were also studied (Figure 6). Generally, larger tumor volumes, surface area, and TD were weakly correlated (*R* ^2^ = 0.088, 0.084, 0.031 for volume, surface area, and TD respectively) with smaller dTICA. There was no statistical significance noted when correlating dTICA across age or gender.

**FIGURE 7:**
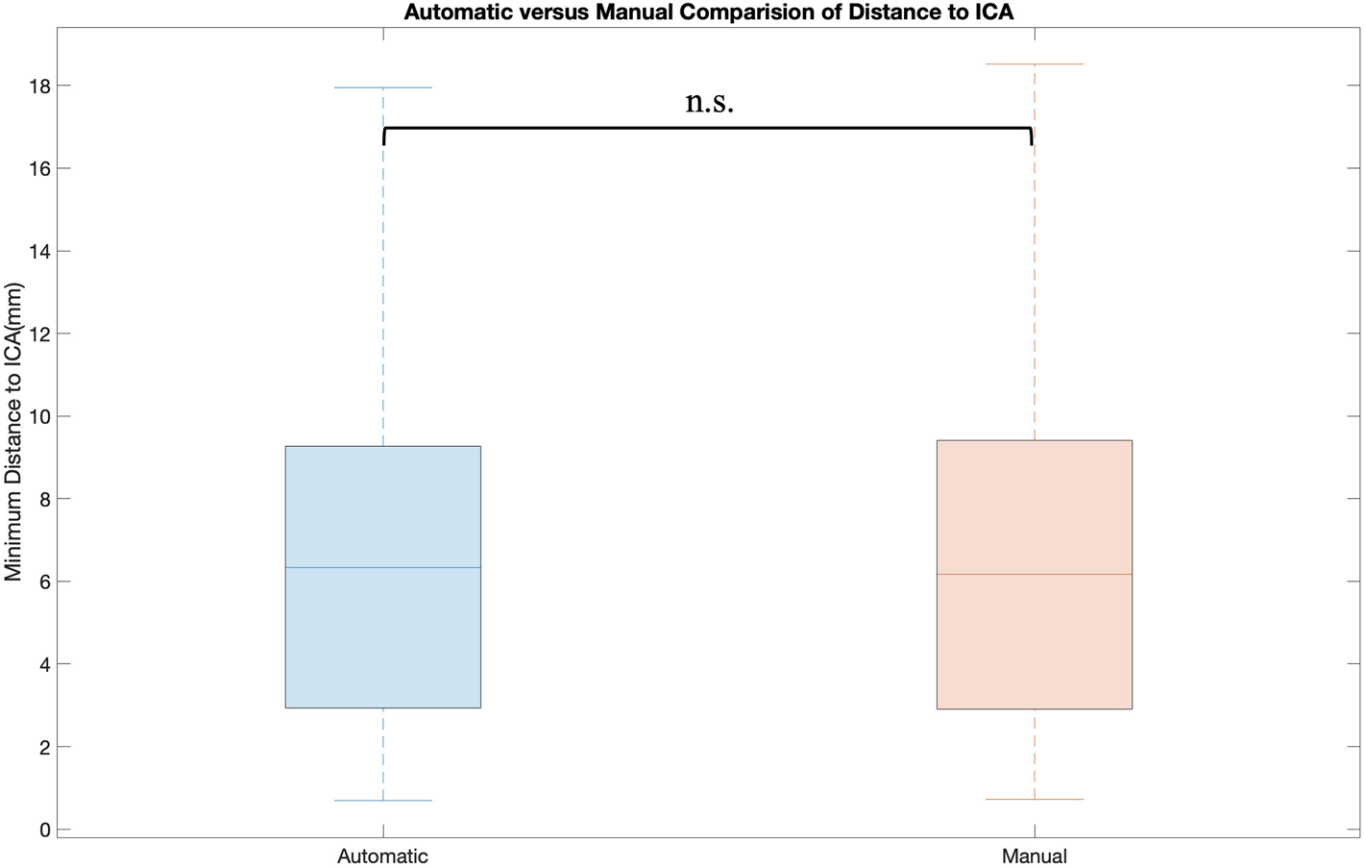
Automatic extraction of distance from tumor to ICA compared to manual measurement from tumor to ICA. [Color figure can be viewed at wileyonlinelibrary.com]

dTICA was also manually calculated from the CT scans and compared to the distance automatically generated from the tool to assess the accuracy of the automated system (Figure 7). There was no statistical difference between manual calculations compared to those generated by the tool (*p* = 0.78).

Table 2 summarizes the tumor characteristics (tumor volume, SA, and TD) of the smaller 60 CT scan dataset by tumor T stage. An ANOVA test was used to assess for statistical significance of volume, SA, and TD differences between all four stages. For all three characteristics studied, there was a statistical significance between the groups (*p* < 0.01 for volume, SA, and TD). Upon further analysis with the two‐sample *t*‐test, the greatest statistical significance was between tumors staged less than or equal to T2 and T3 and greater for all three characteristics studied (*p* < 0.01 for volume, SA, and TD). There was no statistically significant difference between T3 and T4 tumors for volume, SA, or TD. A similar trend was noted between T1 and T2 tumors. Additionally, there was no statistical significance noticed when assessing tumor characteristics across age or gender.

**TABLE 2:** Summary of database by tumor T‐stage.

	T1 tumors	T2 tumors	T3 tumors	T4 tumors	*p*
Volume (mm^3^)					**< 0.001**
Mean	4264	5628	19 887	27 095	
Range	976–11 872	1338–12 034	2226–44 961	4944–77 077	
Surface area (mm^2^)					**< 0.001**
Mean	1744	1874	4584	7855	
Range	548–3920	696–3202	1201–8927	2064–30 772	
Tumor diameter (TD, mm)					**< 0.001**
Mean	36.50	35.82	56.60	63.45	
Range	18.30–64.62	21.73–45.86	30.57–79.81	41.75–118.16	

*Note:* Tumor diameter corresponds to the diameter calculated from the bounding box in the transverse plane (TD). Bold values indicate statistically significant test results.

The location of the minimum distance point (MDP), which represents the closest point on the internal carotid artery (measured as mm away from carotid bifurcation) to the tumor border, was on average 38 mm (range 2.3–95 mm, std. 33 mm). The location of MDP was not correlated with the tumor stage, tumor volume, surface area, or TD, implying that the location of MDP may not be related to tumor characteristics analyzed in this study.

## 4 Discussion

Vascular contraindications to resection of oropharyngeal cancers via TORS include the presence of a retropharyngeal carotid artery or the primary tumor being adjacent to the carotid bulb or ICA [9]. Therefore, it is critical for a clinician to assess the relationship between the ICA and primary tumor volume when assessing the candidacy of a patient for TORS. To improve upon the current manual method for analyzing this feature, this paper presents a DL pipeline that (1) automatically generates segmented 3D models of the tonsillar tumor and ICA from unlabeled CT scans and further extends this pipeline to (2) calculate the minimum distance between these two structures. To the authors' best knowledge, this DL pipeline to automatically extract the distance between the tumor and the ICA is a unique application of the use of DL and medical image processing in head and neck oncology.

Analyzing tumors and vasculature from CT scans can be challenging as they may have noise/artifacts from metal dental implants or patient movement. However, CT remains the gold standard for pre‐operative imaging for head and neck patients. Additionally, nnU‐Net, which is built upon a standard U‐net framework, is well suited to handle these challenges as it offers advantages over traditional DL frameworks with regard to noise and data variability. nnU‐Net has an adaptive pre‐processing pipeline that automatically adjusts hyperparameters like learning rate, intensity normalization, and batch size based on the feature being analyzed, resulting in improved training performance. It also employs robust data augmentation techniques, enhancing its generalizability and noise resilience [23]. Still, artifacts from CT scans, including implants or patient motion, can affect the performance of deep learning models. While nnU‐Net does take some preprocessing steps, including intensity normalization to reduce the impact of artifacts, like radiologists, it relies on clear delineation of the tumor borders for accurate segmentation. The accuracy of the model in these scenarios is usually worse and remains a limitation of deep learning techniques for image segmentation. The authors of the original nnU‐Net also note that it has been rigorously tested on datasets with varying sizes and imaging modalities; it achieves superior segmentation compared to other DL models in most cases [18]. With regard to analyzing DL performance, Muller et al. discuss multiple metrics used to analyze medical imaging segmentations [24]. Per their review, we choose to use average hausdorff distance (AHD) and dice similarity coefficient (DSC) to estimate the surface and volume error, respectively, between the generated inferences and the ground truth. Muller et al. note that these metrics tend to be more widely used compared to other metrics.

Clinically, this tool can be used for pre‐operative evaluation of the ICA relation to the primary oropharyngeal tumor to assist with surgical planning. However, the applications for this tool go beyond pre‐operative assessment and planning. Chan et al. described an augmented reality (AR) system that registers and overlays 3D models of structures including the ICA on the surgical field of view to facilitate image guided TORS [25]. Within this AR system, the proposed tool in this paper could facilitate valuable real‐time calculation between the ICA and operative tool or primary tumor. Additionally, applications of this tool could further be extended to identify other critical structures such as the lingual artery when resecting base of tongue tumors. Other groups have described the importance of measuring the distance to the ICA for procedures such as tonsillectomies or resection of nasopharyngeal carcinomas [26, 27]. Finally, a tool that automatically calculates the dTICA would allow researchers to perform large population studies to identify variations in vascular anatomies and their relation to a primary tonsillar tumor.

We assessed the accuracy of the DL nnU‐Net segmentation pipeline both via the AHD and DSC. Generally, for DL frameworks, an average DSC score > 0.7 is considered acceptable [27]. While the DSC score for ICA segmentation met this requirement, the average DSC score for tonsillar tumors was slightly lower than this threshold (0.67 ± 0.14). For AHD, the threshold for acceptable values varies depending on the application [28]. In our case, we achieved sub 5 mm accuracy for tonsillar tumors that were often on the order of multiple cm^3^ and sub 1 mm accuracy for ICA segmentation, suggesting that the DL framework may still be acceptable. Erroneous segmentations could significantly alter dTICA calculation; such errors could stem from artifacts from CT scans, poor delineation of tumor boundaries, or aberrant anatomy that significantly differs from the scans used to train the network developed in this paper. Given the importance of dTICA, we recommend that segmentation nnU‐Net only be used as a segmentation assistant within a clinical setting. A physician with appropriate training should always verify the accuracy of the segmentation and modify it as appropriate before performing dTICA assessment. We anticipate that with larger datasets and technological enhancements in the field of deep learning, this process will become further refined and fully automated in the future. The nnU‐Net implemented for ICA segmentation successfully met the established performance criteria as determined by the DSC(0.83 ± 0.10).

In addition, we demonstrated that our tool could automatically extract the dTICA and the location (MDP) of the dTICA with the same accuracy as the manual extraction method. This tool, however, relies on the key assumption that the 3D models used for analysis are accurate. For both the deep learning training portion and dTICA calculation, the tumor and ICA models used were manually segmented to generate 3D volumes. The tumor models used were part of the larger public database *Radiomics outcome prediction in Oropharyngeal cancer*, which has been segmented and validated by numerous trained radiologists. Additionally, as mentioned above, the accuracy of the ICA segmentations was verified by senior otolaryngologists (FC, EP).

The trends with regard to tumor shape characteristics (volume, SA, and TD) outlined in Table 2 are generally consistent with AJCC 8th edition tumor staging guidelines [29]. Higher T stage tumors are typically larger and therefore will have greater volume, SA, and TD. The statistically significant difference between tumors less than or equal to T2 and greater than T2 aligns with AJCC guidelines that use 4 cm as a cutoff between T2 (size 2–4 cm) and T3(> 4 cm or extension into the lingual surface of the epiglottis) tumors. The lack of statistical significance for all three of these shape characteristics between T1 (size ≤ 2 cm) and T2 tumors could be due to the way that T staging is measured. According to guidelines, T stage is measured by the greatest dimension in a 2D plane of the tumor, which may not necessarily correlate with volume, SA, and TD. Since the delineation between T3 and T4 staging involves the structures that the tumor extends into, the lack of statistical significance between volume, SA, and TD between these groups makes sense.

As expected, higher tumor stages are typically associated with smaller dTICA. Tumors staged T2 or less had a significantly greater distance between the tumor border and the ICA compared to tumors staged higher than T2. Interestingly, while larger tumor volume, SA, and TD were all associated with smaller dTICA, all three independently were weakly correlated with dTICA using linear regression (*R* ^2^ < 0.8) signifying that independently these are not correlated with dTICA. Theoretically, larger tonsillar tumors (i.e., greater volume, SA, and TD) that expand more circumferentially would be closer to the ICA; however, the weak correlation between the shape characteristics and dTICA suggests that larger tonsillar tumors may not necessarily grow circumferentially. Further analysis with a larger dataset may be needed to explain the association between the directionality of tumor growth and dTICA.

We acknowledge some limitations of the dTICA analysis. The DSC score achieved for the tonsillar tumors was less than the threshold of 0.7 that is typically used as the measurement for acceptable segmentation. As mentioned above, there are numerous reasons for this, including dental artifacts, poor ground truths, rater bias, among others. These errors would likely impact the tumor ICA distance, and as such, we always recommend using tools like these to assist in clinical decision making rather than a sole decision‐making tool. We propose two potential future solutions to improve model performance. One, adding more CT scans segmented by experts to any model can make the model more robust and reduce rater bias. As such, we are planning to increase the size of our database while having multiple providers segment scans. Second, advancements in imaging processing have allowed for better artifact removal and improved segmentation accuracy from CT scans using various deep learning techniques [30]. Groups such as Zilong Li et al. leverage a novel Quad‐Net that utilizes the sinogram, image, and their respective Fourier transforms in a series of convolutional neural networks to eliminate significant streaking artifact from metals [31]. Additionally, Cai et al. implemented Swin Unet3D model that enhances segmentation by combining a convolution and transformer neural network in parallel to effectively capture both local and global dependencies [32]. Such advances could further improve the accuracy of tumor and ICA segmentation from CT images. Future studies could also use more metrics to quantify performance as they become more common. Additionally, the dataset used for the analysis of this tool consisted of only 60 scans, 15 of each tumor T stage. This dataset could be further expanded for future analysis to improve the generalizability of the findings. However, we ensured that it was well balanced pertaining to tumor stage. With regards to demographics, the dataset used, while small, still accurately reflects the overall trends within head and neck cancers, suggesting the findings are generalizable [33]. Finally, while the accuracy of the ICA segmentations was verified by the senior otolaryngologist and data from the *Radiomics outcome prediction in Oropharyngeal cancer* dataset has been validated by numerous trained radiologists, further research needs to be done to quantify the accuracy of these models by validating both the deep learning and dTICA portions of this study with an external dataset. We are currently working to create such a dataset at multiple institutions to achieve external validation of our model. The proposed pipeline should facilitate accurate, timely analysis of the dTICA that can aid in the preoperative evaluation of patients with oropharyngeal squamous cell carcinoma.

## 5 Conclusion

This paper presents the first end‐to‐end deep learning pipeline that allows users to automatically segment tonsillar tumors and ICAs from CT scans and compute the distance between the two structures. Quantifying the distance between the ICA and tonsillar tumor has the potential to aid surgeons in creating optimal preoperative surgical plans, appropriately screen patients before transoral robotic surgery, and integrate intraoperative surgical guidance for identification of critical vascular structures.

## Author Contributions

**Aseem Jain:** project lead, technical and clinical contributions. **Ameen Amanian:** clinical advisor. **Nimesh Nagururu:** technical contributions. **Francis X. Creighton:** clinical advisor. **Eitan Prisman:** senior author, clinical advisor.

## Conflicts of Interest

The authors declare no conflicts of interest.

## Supporting information


**Data S1.** Supporting Information.

## Data Availability

The data that support the findings of this study are available in Oropharyngeal‐Radiomics‐Outcomes at https://www.cancerimagingarchive.net/analysis‐result/oropharyngeal‐radiomics‐outcomes/, reference number 12. These data were derived from the following resources available in the public domain: Oropharyngeal‐Radiomics‐Outcomes, https://www.cancerimagingarchive.net/analysis‐result/oropharyngeal‐radiomics‐outcomes/.
