# Supplementary material for: Deep Learning Pipeline for Automated Assessment of Distances Between Tonsillar Tumors and the Internal Carotid Artery
Source: Head Neck. 2025 Jun 3;47(10):2835–44. doi: 10.1002/hed.28200 (PMC12434563; doi:10.1002/hed.28200)
Supplement: Supplementary file 1 — Data S1. Supporting Information. [file HED-47-2835-s001.docx]

**Tonsil Tumor nnU-Net**

- Network Type: 3D full resolution UNet (PlainConvUNet)

- Image Reader/Writer: SimpleITKIO

- Preprocessing:

- Preprocessor: DefaultPreprocessor

- Normalization: CTNormalization

- Use mask for normalization: False

- Input Spacing: (1.0, 0.488281, 0.488281)

- Original Image Size (after transpose): (170, 512, 512)

- Patch Size: (56, 192, 192)

- Batch Size: 2

- Median Image Size (in voxels): (170.0, 512.0, 512.0)

- Network Architecture:

- Base Number of Features: 32

- Max Number of Features: 320

- Convolutions per Encoder Stage: [2, 2, 2, 2, 2, 2]

- Convolutions per Decoder Stage: [2, 2, 2, 2, 2]

- Number of Pools per Axis: [3, 5, 5]

- Pooling Kernel Sizes:

- Progressive pooling (mostly (2,2,2) with initial (1,2,2))

- Convolution Kernel Sizes:

- First layer: (1,3,3)

- Subsequent layers: (3,3,3)

- Resampling Functions:

- Data: 3rd order spline interpolation (order=3, order_z=0)

- Segmentation: Linear interpolation (order=1, order_z=0)

- Probabilities: Linear interpolation (order=1, order_z=0)

- Loss Handling: Batch Dice Loss enabled

- Cascade Setup: 3D cascade full resolution stage inheriting from a 3D low resolution stage.

**Internal Carotid Artery nnU-Net**

- Network Type: 3D full resolution UNet (PlainConvUNet)

- Image Reader/Writer: SimpleITKIO

- Preprocessing:

- Preprocessor: DefaultPreprocessor

- Normalization: CTNormalization

- Use mask for normalization: False

- Input Spacing: (1.0, 0.488281, 0.488281)

- Original Image Size (after transpose): (200, 512, 512)

- Patch Size: (64, 192, 160)

- Batch Size: 2

- Median Image Size (in voxels): (200.0, 512.0, 512.0)

- Network Architecture:

- Base Number of Features: 32

- Max Number of Features: 320

- Convolutions per Encoder Stage: [2, 2, 2, 2, 2, 2]

- Convolutions per Decoder Stage: [2, 2, 2, 2, 2]

- Number of Pools per Axis: [4, 5, 5]

- Pooling Kernel Sizes:

- Progressive pooling (mostly (2,2,2) with initial (1,2,2))

- Convolution Kernel Sizes:

- First layer: (1,3,3)

- Subsequent layers: (3,3,3)

- Resampling Functions:

- Data: 3rd order spline interpolation (order=3, order_z=0)

- Segmentation: Linear interpolation (order=1, order_z=0)

- Probabilities: Linear interpolation (order=1, order_z=0)

- Loss Handling: Batch Dice Loss enabled

- Cascade Setup: 3D cascade full resolution stage inheriting from a 3D low resolution stage.
